# The importance of cigarillo product characteristics among young adult cigarillo users: Differences by demographics, cigarillo use and other tobacco/substance use behaviors

**DOI:** 10.1371/journal.pone.0265470

**Published:** 2022-04-08

**Authors:** Ollie Ganz, Michelle Jeong, Kevin R. J. Schroth, Mary Hrywna

**Affiliations:** 1 Rutgers Center for Tobacco Studies, Rutgers Biomedical and Health Sciences, New Brunswick, New Jersey, United States of America; 2 Department of Health Behavior, Society and Policy, Rutgers School of Public Health, Piscataway, New Jersey, United States of America; Medical University of South Carolina, UNITED STATES

## Abstract

**Introduction:**

Cigar products, including cigarillos, have increased in popularity in the U.S. and are disproportionately used by young adults. Cigarillo product characteristics can influence consumer perceptions and the appeal of these characteristics can vary by subgroup. The goal of this study was to examine a) product characteristics important to young adult cigarillo users and b) differences based on demographics and cigarillo and other tobacco/substance use behaviors.

**Methods:**

In 2016, a convenience sample of 628 past-year cigarillo users rated the importance of the following cigarillo product characteristics when choosing a cigarillo to smoke tobacco: brand, tobacco filler quality, tobacco wrap quality, flavors, price, package graphic design, and number of cigars in the pack. Differences in mean importance by demographic, cigarillo use and other tobacco/substance use characteristics were analyzed using t-tests and ANOVA tests.

**Results:**

The most important cigarillo product characteristics were price, quality of tobacco wrap, and flavors. The least important was graphic design of packaging. There were differences in importance by demographics and/or cigar and other tobacco/substance use behaviors for all product characteristics. In particular, pack size was rated as more important among current cigarillo users, users of foil pouches (2–3 cigarillos) or cardboard/paper boxes or other packaging styles, and current users of cigarettes, blunts and marijuana. Price was rated as more important among Hispanic/Latino and lower income smokers, and current cigarette and blunt smokers.

**Conclusions:**

Findings suggest cigarillo users prioritize different product characteristics depending on their demographics or smoking behavior. Further research is needed to examine whether various cigar-related policies, such as those that ban flavors or set minimum pack sizes, could impact sub-populations of cigarillo users differently.

## Introduction

As cigarette use and consumption have declined in the United States (U.S.) [1], cigar unit sales have increased over the past two decades [2–5]. Indeed, one recent study found that cigar sales increased from $2.47 billion in 2009 to $3.27 billion in 2020, an average annual percentage change of 2.1% [5]. This increase in cigar sales is mostly driven by cigarillos [5], which are mid-sized cigars that are typically mass-marketed and are the most popular cigar product in the U.S. [6]. In particular, cigarillo use is disproportionately common among young adults [6].

Although the U.S. Food and Drug Administration (FDA) Center for Tobacco Products (CTP) extended its regulatory authority in 2016 to cover all tobacco products, cigarillos and other cigar products are still subject to fewer regulations than cigarettes [7], which has resulted in a diverse cigarillo marketplace. Unlike cigarettes, which have a minimum pack size of 20, cigarillos can be sold as singles and in small pack sizes [8], allowing them to be sold at an affordable price (e.g., 99 cents for a 2-pack). Furthermore, unlike cigarettes, which cannot be sold in characterizing flavors other than menthol, cigarillos are available in a variety of characterizing (e.g., grape, wine) and concept (e.g., Jazz, Swerve) flavors that continue to diversify [2,9]. Although FDA CTP announced in April 2021 their intention to issue a product standard banning flavors in cigars, it is unclear when this will happen [10]. Cigarillos also vary by other product characteristics such as brand, package descriptors and color, and tobacco quality.

It is apparent from the tobacco control literature that cigarillo product characteristics influence consumer perceptions [11–16]. Two qualitative studies among young adult cigarillo smokers found that the variety of flavors, low price, and packaging make cigarillos attractive [11,12]. Several experimental studies reported similar results. For example, one online experiment of young adults found that cigarillo pack flavor descriptors and colors significantly influenced favorable perceptions of the product’s smell and taste and made the product more appealing. Additionally, foil 2-packs were rated as more appealing than box 5-packs [14]. Another experimental study that included young adult little cigar and cigarillo smokers found that price promotions made cigarillo packs more visually appealing and increased interest in the product [13].

Studies have also shown that reasons for cigarillo use and preferences for different cigarillo product characteristics, such as pack size, brand and flavors, differ across population subgroups [17–20]. For example, Dunn et al. found differences in reasons for cigarillo use among cigarillo users by race, such that a greater proportion of Black ever cigarillo users reported affordability, reduced perceived harm and appealing advertising as reasons for use, compared with White users [17]. Additional research using data from the Population Assessment of Tobacco and Health Study found that smaller cigar pack sizes were preferred among younger and female Black & Mild cigar smokers [18].

However, gaps in the evidence base persist regarding the context in which these preferences for cigar product characteristics arise. Specifically, the preponderance of research has either focused on differences based on a single demographic characteristic (e.g., race) [17] or only examined demographic differences for one product feature, such as pack size, brand or flavor [18–20]. Furthermore, there is a paucity of research on how preferences for cigar product characteristics differ based on cigar and other tobacco use characteristics. This study begins to address these gaps and examines product characteristics important to young adult cigarillo users. Based on a secondary data analysis of an online survey of past year cigar users aged 18–34, we explored preferences for a comprehensive list of cigarillo product characteristics, examining how such preferences differed by demographic and tobacco/substance use characteristics.

## Materials and methods

### Participants and procedures

These data were derived from an experiment designed to assess product preferences, perceptions and behaviors among young adults and were collected using Amazon Mechanical Turk (MTurk) in 2016 [15]. Eligibility criteria included a) being 18–34 years old and b) having used a cigar (i.e., any type of cigar or cigarillo or smoked a blunt) in the past 12 months. Participants provided consent online prior to participation in the study. The initial survey sample included 1,260 participants. The Rutgers Biomedical and Health Sciences Institutional Review Board approved all research procedures for this study.

Once a participant was deemed eligible, consented to participate, and began the survey, they were asked separately about past 12-month use of each type of cigar product (i.e., traditional cigars, filtered cigars and cigarillos). To reduce potential misclassification (e.g., selecting that you smoke a cigarillo when you really smoke a filtered cigar), a picture of the corresponding product, as well as brand examples, accompanied the past 12-month use question for each product. Past-year cigarillo smokers were then asked, “Which of the following best describes how you have used cigarillos in the past 12 months,” 1) I have only used cigarillos to smoke tobacco, 2) I have only used cigarillos as a blunt to smoke marijuana and 3) I have used cigarillos **both** to smoke tobacco and as a blunt to smoke marijuana. Those who responded that they only used cigarillos to smoke tobacco or both tobacco and blunts were included in the analytic sample (n = 628). Those who reported smoking cigarillos only as blunts were not included the analysis.

Eligible participants completed survey measures related to cigar use preferences and behaviors, other tobacco and substance use behaviors and demographics.

### Measures

#### Cigarillo product characteristics

The primary variable of interest, importance of different cigarillo characteristics, was assessed with the following question: “When choosing a cigarillo **to smoke tobacco**, how important are the following product characteristics?” Respondents then rated the following characteristics from 1 (very important) to 4 (not at all important): brand, quality of the tobacco filler, quality of the tobacco wrap, flavors, price, graphic design of the packaging and number of cigars in the pack. For the analysis, values were reverse coded such that a higher score indicated greater importance.

#### Demographic characteristics

All participants were asked to report the following demographic characteristics: gender (male or female), age (18–24 or 25–34), race (White, Black, or Other), ethnicity (Hispanic/Latino or non-Hispanic/Latino), educational attainment (12^th^ grade/GED or less or some college or more) and annual income ($30,000 or less or more than $30,000).

#### Cigarillo behaviors

Current cigarillo users were defined as past 12-month users who reported currently using cigarillos to smoke tobacco every day or some days. Flavored cigarillo use was assessed among all past-year users with the question: “Are the cigarillos that you use to smoke tobacco flavored?” (yes/no). Cigarillo brand preference was assessed with the following question: “What brand of cigarillos do you use most often **to smoke tobacco**?” Answer choices included: I do not have a usual brand, Black & Mild, Swisher Sweets, White Owl, Dutch Masters, Garcia y Vega, Backwoods, Zig Zag, Phillies, Al Capone and other.

Past-year cigarillo users were also asked about their specific cigarillo and packaging preferences. Users were asked, “Select the packaging style that is most similar to the cigarillos that you use **to smoke tobacco**: 1) a foil pouch that usually holds 2 to 3 cigarillos, 2) a single cigarillo wrapped in foil or plastic, 3) a cardboard or paper box or 4) another packaging style not shown here.” For analyses, those who selected “another packaging style not shown here” (n = 11) were combined with those who selected “a cardboard or paper box,” creating a new category called “cardboard/paper box/another style.”

#### Other tobacco and substance use behaviors

Current cigarette use was defined as having smoked at least 100 cigarettes in one’s lifetime and currently smoking every day or some days [21]. Current use of other tobacco products was defined as every day or someday use of at least one of the following: a) smokeless tobacco such as dip, snus or chew, b) hookah, or c) electronic cigarettes or another vaping product. Current marijuana use was defined as every day or someday use of marijuana. Current blunt use (i.e., when some or all of the tobacco is removed from the cigar and replaced with marijuana), was assessed among past-year blunt users with the following question: “Do you currently use cigarillos as blunts to smoke marijuana every day, some days, rarely or not at all?” Those who selected every day or some days were categorized as current users. Those who selected rarely, not at all or reported not using blunts in the past year were categorized as non-current blunt users.

### Data analyses

First, we used descriptive statistics to characterize the analytic sample in terms of demographics, cigar use behaviors and preferences, and use of other substances and tobacco products. Next, we calculated the mean importance of each cigarillo product characteristic overall, as well as by demographics, cigar use behaviors and preferences, and use of other substances and tobacco products. We then examined differences in mean importance using t-tests for variables with two categories and one-way ANOVA tests for variables with more than two categories. For significant ANOVA tests, we ran post-hoc pairwise comparisons using Bonferroni adjustment to identify which categories differed from one another in terms of product importance. All statistical tests were conducted in Stata/MP 16.1 [22].

## Results

### Overall sample characteristics

Table 1 presents the demographic characteristics of the analytic sample, and importance of each cigarillo product characteristic both overall and by demographics. Briefly, the sample was mostly male (68.70%), ages 25–34 (66.08%), White (78.18%), non-Hispanic (88.69%), completed some college or more (88.22%) and reported an annual income of $30,000 or less (59.02%). Table 2 presents importance of each cigarillo product characteristic by cigarillo and other tobacco/substance use behaviors. While everyone in the analytic sample was a past-year cigarillo user, the majority were not current users (67.68%). The most popular cigarillo brands were Black & Mild (47.61%) and Swisher Sweets (25.48%). The majority of the sample used flavored cigarillos (63.85%) and cigarillos sold in a foil pouch with 2–3 cigarillos (39.94%) or in a cardboard or paper box or other style (33.71%). Slightly less than half of the sample reported current cigarette use (43.31%) and current marijuana use (45.06%), while approximately one-third of the sample reported current use of other tobacco products (34.24%) and blunts (29.14%).

**Table 1:** Mean importance of cigarillo product characteristics by demographics.

	Overall sample characteristics of past-year cigarillo users (N = 628)	Brand (N = 626)	Quality of the tobacco filler (N = 626)	Number of cigars in pack (N = 628)	Quality of the tobacco wrap (N = 627)	Flavors (N = 628)	Price (N = 621)	Graphic design on packaging (N = 628)
	% (n)	M (SD)	M (SD)	M (SD)	M (SD)	M (SD)	M (SD)	M (SD)
**Overall**		2.92 (0.86)	2.95 (0.93)	2.93 (0.86)	3.20 (0.83)	3.17 (0.88)	3.38 (0.76)	1.82 (0.81)
**Demographics**								
Gender								
Male	68.70 (432)	2.95 (0.85)	2.96 (0.93)	2.90 (0.84)	3.21 (0.81)	**3.11 (0.90)**	3.34 (0.77)	1.81 (0.80)
Female	31.21 (196)	2.87 (0.86)	2.91 (0.95)	2.98 (0.89)	3.17 (0.87)	**3.31 (0.82)**	3.46 (0.71)	1.85 (0.84)
Age								
18–24	33.92 (213)	**3.05 (0.84)**	**2.81 (0.98)**	2.96 (0.89)	3.22 (0.82)	**3.30 (0.82)**	3.43 (0.75)	1.80 (0.87)
25–34	66.08 (415)	**2.86 (0.87)**	**3.01 (0.91)**	2.91 (0.84)	3.19 (0.83)	**3.10 (0.90)**	3.35 (0.76)	1.83 (0.78)
Race								
White	78.18 (491)	**2.87 (0.86)** ^**a**^	2.92 (0.92)	2.90 (0.85)	3.18 (0.81)	3.20 (0.87)	3.36 (0.76)	1.81 (0.80)
Black	11.31 (71)	**3.26 (0.85)** ^**b**^	3.11 (0.96)	3.11 (0.89)	3.28 (0.90)	3.07 (0.95)	3.39 (0.82)	1.90 (0.91)
Other	10.51 (66)	**2.97 (0.84)** ^**a,b**^	2.97 (0.99)	2.95 (0.90)	3.26 (0.86)	3.06 (0.89)	3.48 (0.64)	1.79 (0.75)
Ethnicity								
Hispanic/Latino	11.31 (71)	3.01 (0.82)	2.97 (1.00)	3.11 (0.95)	3.30 (0.87)	3.11 (0.84)	**3.56 (0.63)**	1.91 (0.84)
Non-Hispanic/Latino	88.69 (557)	2.91 (0.87)	2.94 (0.93)	2.90 (0.84)	3.18 (0.82)	3.18 (0.89)	**3.35 (0.77)**	1.81 (0.81)
Educational attainment								
12^th^ grade/GED or less	11.78 (74)	2.89 (0.97)	2.99 (0.96)	2.96 (0.91)	**3.45 (0.69)**	**3.43 (0.72)**	3.54 (0.65)	1.88 (0.72)
Some college or more	88.22 (554)	2.92 (0.85)	2.94 (0.93)	2.92 (0.85)	**3.16 (0.84)**	**3.13 (0.89)**	3.36 (0.77)	1.81 (0.82)
Annual income								
$30,000 or less	59.02 (360)	2.90 (0.87)	2.94 (0.94)	2.97 (0.86)	**3.26 (0.77)**	3.22 (0.84)	**3.44 (0.72)**	1.77 (0.78)
More than $30,000	40.98 (250)	2.95 (0.85)	2.94 (0.95)	2.85 (0.85)	**3.12 (0.89)**	3.09 (0.06)	**3.27 (0.80)**	1.88 (0.84)

M = mean; SD = standard deviation.

For variables with 2 levels, bold indicates statistical significance at p < .05, obtained from t-tests or one-way ANOVA.

For variables with 3 or more levels, superscripts (e.g., ^a, b^) indicate statistical significance at p < .05, such that means with different superscripts are significantly different from each other. Means with the same superscript are not significantly different from each other.

Values range from 1–4, with a higher number indicating greater importance.

**Table 2:** Mean importance of cigarillo product characteristics by tobacco and other substance use characteristics.

	Overall sample characteristics of past-year cigarillo users (N = 628)	Brand (N = 626)	Quality of the tobacco filler (N = 626)	Number of cigars in pack (N = 628)	Quality of the tobacco wrap (N = 627)	Flavors (N = 628)	Price (N = 621)	Material of packaging (N = 627)	Graphic design on packaging (N = 628)
	% (n)	M (SD)	M (SD)	M (SD)	M (SD)	M (SD)	M (SD)	M (SD)	M (SD)
**Cigarillo use behaviors**									
Current cigarillo use									
Yes	32.32 (203)	**3.08 (0.89)**	**3.09 (0.97)**	**3.13 (0.83)**	**3.46 (0.80)**	3.13 (0.88)	3.36 (0.81)	**2.07 (0.95)**	1.88 (0.89)
No	67.68 (425)	**2.85 (0.84)**	**2.88 (0.91)**	**2.83 (0.86)**	**3.07 (0.83)**	3.25 (0.88)	3.39 (0.73)	**2.87 (0.80)**	1.79 (0.77)
Cigarillo brand preference									
No brand preference	7.48 (47)	**2.28 (0.80)** ^**a**^	3.11 (0.89)	**2.70 (0.95)** ^**a,b**^	**3.15 (0.83)** ^**a,b**^	**3.04 (0.95)** ^**a,b**^	3.35 (0.77)	2.02 (0.85)	1.87 (0.82)
Black & Mild	47.61 (299)	**2.96 (0.87)** ^**b**^	2.98 (0.90)	**2.87 (0.87)** ^**a,b**^	**3.14 (0.82)** ^**a**^	**3.16 (0.91)** ^**a,b**^	3.37 (0.75)	1.90 (0.82)	1.77 (0.76)
Swisher Sweets	25.48 (160)	**2.94 (0.85)** ^**b**^	2.82 (0.95)	**3.11 (0.79)** ^**c**^	**3.14 (0.90)** ^**a,b**^	**3.31 (0.80)** ^**a**^	3.49 (0.72)	1.96 (0.93)	1.89 (0.89)
White Owl	7.96 (50)	**2.94 (0.87)** ^**b**^	2.74 (1.05)	**2.86 (0.95)** ^**a,b,c**^	**3.42 (0.73)** ^**a,b**^	**3.28 (0.78)** ^**a,b**^	3.18 (0.77)	1.84 (0.87)	1.64 (0.72)
Other	11.46 (72)	**3.15 (0.74)** ^**b**^	3.10 (0.96)	**2.97 (0.77)** ^**a,b,c**^	**3.46 (0.71)** ^**b**^	**2.92 (0.90)** ^**b**^	3.34 (0.81)	2.06 (0.84)	1.97 (0.850
Flavored cigarillo use									
Yes	63.85 (401)	2.94 (0.86)	**3.06 (0.90)**	2.91 (0.83)	**3.15 (0.85)**	**3.38 (0.68)**	3.36 (0.76)	1.94 (0.85)	1.79 (0.81)
No	36.15 (227)	2.90 (0.86)	**2.88 (0.95)**	2.96 (0.90)	**3.31 (0.78)**	**2.79 (1.05)**	3.41 (0.75)	1.93 (0.86)	1.87 (0.83)
Cigarillo packaging preference									
Foil pouch (2–3 cigarillos)	39.94 (250)	2.96 (0.83)	**2.77 (1.01)** ^**a**^	**3.09 (0.79)** ^**a**^	3.25 (0.82)	**3.35 (0.79)** ^**a**^	3.42 (0.75)	1.96 (0.89)	1.86 (0.87)
Single cigarillo	26.36 (165)	2.89 (0.92)	**3.07 (0.90)** ^**b**^	**2.60 (0.96)** ^**b**^	3.23 (0.82)	**3.09 (0.94)** ^**b**^	3.39 (0.74)	1.97 (0.87)	1.78 (0.76)
Cardboard/paper box/another style	33.71 (211)	2.90 (0.86)	**3.04 (0.84)** ^**b**^	**2.99 (0.79)** ^**a**^	3.11 (0.83)	**3.01 (0.90)** ^**b**^	3.31 (0.77)	1.88 (0.80)	1.81 (0.79)
**Other tobacco and substance use**									
Current cigarette use									
Yes	43.31 (272)	2.98 (0.88)	2.99 (0.95)	**3.01 (0.87)**	**3.27 (0.82)**	**3.25 (0.87)**	**3.47 (0.74)**	2.00 (0.86)	1.82 (0.85)
No	56.69 (356)	2.88 (0.85)	2.91 (0.92)	**2.86 (0.85)**	**3.14 (0.83)**	**3.10 (0.89)**	**3.31 (0.76)**	1.89 (0.86)	1.82 (0.78)
Current use of other tobacco products (smokeless, hookah, ecigs)									
Yes	34.24 (215)	2.99 (0.88)	3.00 (0.96)	3.01 (0.86)	3.25 (0.80)	3.24 (0.80)	3.35 (0.78)	**2.10 (0.92)**	1.91 (0.91)
No	65.76 (413)	2.89 (0.85)	2.92 (0.92)	2.89 (0.86)	3.17 (0.84)	3.13 (0.92)	3.39 (0.74)	**1.85 (0.81)**	1.77 (0.75)
Current blunt use									
Yes	29.14 (183)	3.03 (0.88)	**2.99 (0.91)**	**3.10 (0.85)**	**3.40 (0.78)**	3.21 (0.86)	**3.50 (0.72)**	1.98 (0.93)	1.84 (0.88)
No	70.86 (445)	2.88 (0.85)	**2.83 (0.99)**	**2.86 (0.85)**	**3.11 (0.83)**	3.15 (0.89)	**3.33 (0.76)**	1.92 (0.83)	1.82 (0.78)
Current marijuana use									
Yes	45.06 (283)	2.94 (0.87)	**2.84 (0.96)**	**3.03 (0.86)**	**3.35 (0.76)**	3.20 (0.85)	3.43 (0.73)	1.98 (0.91)	1.86 (0.83)
No	54.94 (345)	2.91 (0.86)	**3.03 (0.90)**	**2.85 (0.85)**	**3.07 (0.86)**	3.14 (0.90)	3.33 (0.77)	1.90 (0.81)	1.79 (0.79)

M = mean; SD = standard deviation.

For variables with 2 levels, bold indicates statistical significance at p < .05, obtained from t-tests or one-way ANOVA.

For variables with 3 or more levels, superscripts (e.g., ^a, b^) indicate statistical significance at p < .05, such that means with different superscripts are significantly different from each other. Means with the same superscript are not significantly different from each other.

Values range from 1–4, with a higher number indicating greater importance.

### Differences in importance of cigarillo product characteristics

Among the overall analytic sample, the most important cigarillo product characteristics were price (mean (M): 3.38; range 1–4), quality of tobacco wrap (M: 3.20), and flavors (M: 3.17) (Table 1). The lowest scored characteristic in terms of importance was graphic design of packaging (M: 1.82).

#### Brand

Young adults (ages 18–24) and Black respondents rated brand as being more important compared with older young adults (ages 25–34) and White respondents, respectively. Brand was also rated as more important among current cigarillo users (versus non-current users) and users of specific brands compared with those with no brand preference.

#### Quality of the tobacco filler

Older young adults (ages 25–34) rated quality of the tobacco filler as being more important compared with young adults (ages 18–24). Quality of the tobacco filler was more important among current cigarillo users and flavored cigarillo users, compared with non-current users and non-flavored users, respectively. Those who preferred single cigarillos and cigarillos sold in a cardboard/paper box or other packaging reported quality of the tobacco filler as being more important compared with those who preferred foil pouches (2–3 cigarillos). Current blunt users were also more likely to report quality of the tobacco filler as being more important compared with non-current blunt users, whereas current marijuana users rated quality of the tobacco filler as being less important compared with non-current marijuana users.

#### Number of cigars in the pack

Current cigarillo users reported number of cigarillos in the pack as being more important compared with non-current users. Users of Swisher Sweets rated number of cigarillos in the pack as more important compared with users of Black & Mild cigarillos and those with no brand preference. Respondents who used single cigarillos reported number of cigarillos in the pack as being less important compared with those who preferred foil pouches (2–3 cigarillos) or cardboard/paper boxes or other packaging styles. Current users of cigarettes, blunts, and marijuana all reported number of cigarillos in the pack as being more important compared with non-current users of those respective products.

#### Quality of the tobacco wrap

Those with lower levels of education and lower annual income rated quality of the tobacco wrap as more important compared to those with higher levels. Quality of the tobacco wrap was rated as more important among current cigarillo users versus non-users, and less important among flavored cigarillo users, compared with non-flavored users. Users of Black & Mild rated quality of the tobacco wrap as being less important compared with users of “other” brand(s). Current users of cigarettes, blunts, and marijuana all reported quality of the tobacco wrap as being more important compared with non-current users of those respective products.

#### Flavors

Females, young adults (ages 18–24), those with lower levels of education and current cigarette smokers rated flavors as being more important compared with males, older young adults (ages 25–34), those with higher levels of education and non-current cigarette smokers, respectively. Flavors were rated as more important among Swisher Sweets smokers compared with those that used “other” brand(s). Flavored cigarillo users and users of foil pouches (2–3 cigarillos) rated flavors as more important compared with non-flavored cigarillo users and users of single cigarillos and cardboard/paper boxes or other packaging styles, respectively.

#### Price

Price was rated as more important among Hispanic/Latino and lower income respondents, compared with non-Hispanic/Latino and higher income respondents, respectively. Current cigarette and blunt users rated price as more important compared with non-users of these products.

#### Graphic design of packaging

There were no differences in importance of graphic design of the packaging by demographic, cigarillo use or other tobacco/substance use characteristics.

## Discussion

Using data from a national convenience sample of past-year cigarillo users, this study examined the importance of various cigarillo product characteristics, including brand, quality of the tobacco filler, number of cigars in the pack, quality of the tobacco wrap, flavors, price and graphic design on the packaging. This is one of the first studies to identify the importance of different product characteristics in relation to one another, with findings showing that price, flavors, and quality of the tobacco wrap were rated as most important among the sample. These findings align with other research showing that flavors and price are important characteristics to cigarillo smokers [11,12,17,20,23].

Our study also identified differences in importance of product characteristics, based on demographic, cigarillo use and other tobacco/substance use characteristics. Findings showed that brand was rated as more important among Black smokers compared with White smokers, which may reflect targeted marketing efforts toward the Black community by cigarillo companies [24–26]. Brand was also rated as more important among young adults (ages 18–24) compared with older young adults. It is possible that to the general public, the concept of a brand encompasses various superficial aspects of the product and its marketing, including advertisements and product packaging. As such, young adults, who are more susceptible to tobacco industry marketing [27] and are frequent targets of tobacco industry marketing efforts [27], may be rating brand as important for this reason. This finding aligns with one study showing that branding was one of the most attractive cigar packaging features among adolescents and young adults [12]. Future qualitative research could provide insight into how and why brand is an important characteristic among young adults, to better inform regulatory decisions surrounding cigarillo product branding and marketing towards young people.

Interestingly, findings not only revealed no differences in the importance of the graphic design of the cigarillo pack but also low ratings overall and across all sub-populations. Given that prior experimental studies show evidence for the impact of cigarillo package color and design on young adults’ perceptions [14–16], we speculate that this discrepancy between prior experimental findings and self-reported survey data from this study may be due to people’s unwillingness to admit their susceptibility to marketing (and their belief that while everyone else may be influenced, they are not–a phenomenon known as the third-person effect) [28]. Thus, participants may be rating package design as low in importance, while compensating for this by rating brand–which we speculate above as potentially being perceived as including elements such as package design–as high in importance. Experimental studies that measure implicit appeal may be more appropriate for understanding the appeal of the graphic design of packaging.

Several differences that we identified in the importance of flavors are consistent with other research. For example, we found that flavors were more important to females versus males [20,29] and younger adults versus older young adults [20]. Findings also showed that users of foil pouches (2–3 cigarillos) rated flavors as more important compared with users of other packaging styles likely because we often see the most flavor innovation among 2–3 packs [9]. Therefore this may explain or reflect overall increasing popularity in both flavors and small pack sizes [5,30].

Current cigarette smokers, who comprised about 43% of the sample, rated pack size, quality of tobacco wrap, flavors, and price as more important compared with non-current cigarette smokers. The high prevalence of cigarette smoking among the sample overall aligns with research showing high rates of poly tobacco use among young adults [6]. Our finding in regards to pack size is consistent with findings from a recent study of Black & Mild smokers, which showed that users of smaller pack sizes were more likely to be cigarette smokers compared with users of larger pack sizes [18], suggesting that dual users have preferences regarding cigarillo pack size. Dual users may purchase smaller cigarillo packs, which are cheaper than a pack of cigarettes, to keep costs down. This would also explain the greater importance of price among current versus non-current cigarette smokers.

When assessing cigarillo use, it is difficult to disentangle use of the product for smoking tobacco versus smoking blunts [31], a behavior that is particularly common among young adults [11,32,33]. This is an important distinction since research suggests that individuals may prefer different cigarillo product characteristics depending on how they intend to use the cigarillo (i.e., for tobacco or for blunt use) [11,32]. For example, Giovenco et al. found that young adults used different cigarillo brands for different purposes; Swisher Sweets were perceived as being used to smoke blunts, while Black & Milds were perceived as being used to smoke tobacco [11]. In an attempt to address this, our study asked specifically about product characteristics that are important to users when using a cigarillo to smoke tobacco. Following this question, blunt users were asked the same question, but with language specifying product characteristics for using a cigarillo as a blunt to smoke marijuana (this question was not included in the present analyses). As such, we were cautious to attribute any findings from this study to blunt or marijuana use.

Although our survey attempted to tease apart the importance of product characteristics of cigarillos for the purposes of smoking tobacco versus marijuana, the high prevalence of blunt and marijuana use in our analytic sample and certain findings related to product characteristics suggest that blunt use may still have played a role in how individuals answered this question. Specifically, the high rating among the overall analytic sample given to the importance of the quality of tobacco wrap may be related to blunt use, since the cigarillo wrap is a central component of a blunt. Additionally, current blunt and marijuana users rated number of cigars in the pack as more important compared with non-users, as did users of foil pouches (2–3 cigarillos), compared with those who usually buy singles. This aligns with qualitative research on blunt use, which suggests that the 2–3 pack resealable, foil pouches are preferred among blunt users, as they can be used to store and conceal marijuana [11]. It is unclear why blunt and marijuana users had different perspectives on the quality of tobacco filler, with current blunt users rating the quality of the tobacco filler higher compared with non-current blunt users and current marijuana users rating tobacco filler quality lower relative to non-current marijuana users. These differences could be related to the fact that blunt users may actually smoke some of the tobacco filler, whereas marijuana users may not necessarily use any filler, depending on how they consume marijuana (e.g. pipes). More research is needed to understand how smokers use cigarillos for different purposes, including for tobacco, marijuana, or both, and how to effectively measure use for these different purposes.

This study has limitations. First, this study used a convenience sample and therefore may not be generalizable to the young adult, cigarillo smoking population in the U.S. Additionally, data were collected in 2016 and therefore may not entirely reflect the current tobacco marketplace, which is always in flux with the continual emergence of new cigarillo brands, styles and flavors [2,9]. Additionally, the question used to assess importance of different cigarillo product characteristics did not include definitions or examples of the different characteristics (e.g., tobacco filler, tobacco wrap), which may have led to respondents interpreting the question differently. More research is needed to improve survey measurement of different cigarillo characteristics. Finally, this study relies on self-reported data which may be prone to biases. However, our findings provide important preliminary data and insight that can be a foundation for future research.

## Conclusions

FDA CTP has the authority to regulate cigarillos and how they are marketed to consumers but needs a robust body of evidence to do so. Although research shows that cigarillo packaging and other characteristics impact consumer perceptions and behaviors, there is still a void in our knowledge base. Our study fills a gap in the tobacco regulatory science literature by highlighting characteristics that are important to cigarillo smokers and identifying key differences across demographics, cigarillo use and other tobacco/substance use behaviors. Further research is needed to understand how the importance of different product characteristics influence initiation and predict cigar use over time. Importantly, our findings suggest that various cigar-related policies could impact sub-populations of cigarillo users differently (e.g., a flavor ban may influence younger adult and female cigarillo smokers in particular). Future research is needed to understand how different cigar-related policies, such as those that ban flavors or set minimum pack sizes, would impact different groups.
